# A Delphi study for exploring nutritional policy priorities to reduce prevalence of non-communicable diseases in Islamic Republic of Iran

**DOI:** 10.15171/hpp.2019.33

**Published:** 2019-08-06

**Authors:** Hassan Eini Zinab, Naser Kalantari, Alireza Ostadrahimi, Jafar Sadegh Tabrizi, Samira Pourmoradian

**Affiliations:** ^1^Department of Community Nutrition, National Nutrition and Food Technology Research Institute, Faculty of Nutrition Sciences and Food Technology, Shahid Beheshti University of Medical Sciences, Tehran, Iran; ^2^Nutrition Research Center, School of Nutrition, Tabriz University of Medical Sciences, Tabriz, Iran; ^3^Department of Health Service Management, Tabriz Health Service Management Research Centre, School of Health Management and Medical Informatics, Tabriz University of Medical Sciences, Tabriz, Iran; ^4^Food & Nutrition Policy, Student Research Committee, Department of Community Nutrition, National Nutrition and Food Technology Research Institute, Faculty of Nutrition Sciences and Food Technology, Shahid Beheshti University of Medical Sciences, Tehran, Iran

**Keywords:** Nutritional policy, NCDs, Iran

## Abstract

**Background:** Identifying and prioritizing the most appropriate policies for enhancing nutritional habits are vital for the prevention and control of non-communicable diseases (NCDs). This study was conducted to prioritize the nutritional policies in Iran.

**Methods:** A cross-sectional survey applying the two-round Delphi technique was used to prioritize policy options in preventing the burden of NCDs. In the first round, the experts in health and nutrition policy were asked to prioritize 21 policy options on a 5-point Likert scale. After analyzing the first-round questionnaire, the highest mean and lowest dispersion index were calculated as an indicator of high-priority options. In the second round, the policy options suggested by the participants were added to the second-round questionnaire. Finally, the questionnaires were sent to all the participants in case they desired to change their opinions.

**Results: ** The expert achieved consensus on "principles of healthy eating" courses in the curriculum of students as a high-priority policy option. In this regard, "promoting community education and customizing healthy food choice" was the next high priority policy option. On the other hand, the lowest policy priority option was "sending free/low-price healthy drinks at home". The three high priority policy categories were reformulating the content of food, enhancing the consumers’ knowledge, and food labeling, respectively.

**Conclusion:** Our findings showed that reformulation, food promotion, and food labeling had the highest priorities for preventing NCDs in Iran. Although food provision policies were cost effective in developing countries such as Iran, it is essential to provide sub-structures for the reformulation of food products.

## Introduction

Non-communicable diseases (NCDs) are responsible for approximately two-third of the total mortality cases worldwide, of which 82% has been reported in low- and middle-income countries. Annually, a substantial amount of these countries’ income is spent on controlling NCDs.^1^ According to the World Health Organization (WHO), most of the mortality and morbidity cases in the world are connected to NCDs, specifically cardiovascular disorders (CVD), cancer, diabetes, and chronic obstructive pulmonary diseases.^2^ It has been anticipated that, until 2020, NCDs will be responsible for 75% of the total cases of mortality and morbidity. Similarly, in Iran, more than 76% of the total cases of mortality and morbidity are related to NCDs. In 2013, NCDs were responsible for 7 million years of living with a disability in Iran. In the same year, 236 000 deaths occurred due to NCDs; this number had increased up to 14.5% in comparison with the preceding two decades.^3^

Since 2016, NCDs were considered as a great threat for achieving the United Nations’ Millennium and Sustained Developmental Goals. It is predicted that the rapid increase in NCDs leads to increased health care costs, which in turn would hamper the governments’ efforts in battling poverty in low-income countries.^3-5^ In 2011, the representatives of 190 countries agreed upon the 2013-2020 Global Action Plan for preventing and controlling the prevalence of NCDs.^2^ The WHO has designed and implemented different policies and programs in various countries for improving health and preventing NCDs.

These policies are divided into upstream and downstream interventions. The ‘downstream’ policies aim to promote healthy eating through behavioral interventions at individual levels, while the ‘upstream’ policies focus on providing healthier environments. The upstream policies could be categorized according to the 4Ps (price, place, promotion, and product) social marketing framework. In this framework, food price refers to policies affecting prices through taxes, subsidies, or economic incentives. Food promotion refers to policies such as advertising/marketing particularly for children, media campaigns, and health education. Food provision is about policies implemented in specific settings such as schools or workplaces. Food composition refers to the reformulation or elimination of some nutrients from food, food labeling of food products, and menu labeling of served food in stores/restaurants. Food supply chain, trade, and investment include legislation or regulation affecting production policies or supply-chain logistics (Table 1).^6^ In developed countries, successful policies are typically accomplished. For example, the national program commenced for reducing salt in food products and promoting consumer’s general information of Finland and England are two of the most successful policies for reducing salt intake. Thirty years after the beginning of this program, salt intake was reduced as much as one-third leading to a 10-mmHg reduction in systolic and diastolic blood pressure.^7,8^ Although the global and national capacity for implementing these interventions and programs have been inefficient in most developing countries, a review study claimed that, among the 116 developing countries with low or middle income, 29 countries had no policy for confronting chronic NCDs.^9^ Therefore, in these countries, lack of comprehensive policies, efficient and alternative strategies, along with consequences of the localization of successful policies are among the major concerns of health policymakers.

In Iran, the most important risk factors for NCDs include unhealthy dietary habits, smoking, low physical activity, and alcohol consumption.^3^ In Iran, similar to other countries, Ministry of Health and Medical Education (MoHME) has also insisted on developing a national committee for preventing and controlling NCDs and their related risk factors. In this national committee, inner-section objectives are to reduce early death caused by NCDs (25%), insufficient physical activity (20%), alcohol consumption (10%), salt/sodium intake (30%), tobacco use (30%), prevalence of hypertension (25%), preventing further increases in obesity, and diabetes prevalence. In addition, achieving 100% coverage of medications necessary for treating NCDs and at least 70% coverage of other essential drugs, as well as providing counseling for preventing CVD, brain strokes, and legislating against trans fatty acids in edible oils and food products are among the other goals MoHME hopes to achieve.^3^

In Iran, although several policy approaches have been designed and implemented for tackling NCDs, their effectiveness is not usually tested before implementation. These options are broad and potentially confusing, with continuing uncertainties about which interventions are most effective in different ethnic or socioeconomic subgroups. To inform their decisions, policymakers need clear evidence on the potential population benefits as well as the ease and cost of implementation. To help policymakers in this field, it might be helpful to adopt evidence-based policymaking approaches of other countries used for prioritizing efficient and effective policies, based on the opinions of experts and stakeholders of food and nutrition sector. In this way, it would be possible to introduce the most efficient policy options and in accordance with the current socioeconomic status and high prevalence’s noncommunicable diseases in Iran. This study is aimed to help policymakers determine strategies and operational plans to reduce the burden of NCDs in Iran.

## Materials and Methods

We conducted a two-round Delphi study among health managers and other experts in health and nutrition policymaking for answering the main research question: “Which of these policy options are appropriate and have high priority of action in Islamic Republic of Iran?”

Participants consisted of stakeholders and pundits of governmental and private sections and non-profit organizations, which were identified using the Walt-Gilson policy triangle.^10^ This framework put policy content, process, and context on the three corners of the triangle and policy actors in the center. Considering this framework, we mapped stakeholders involved in nutrition, food policymaking, and process in Islamic Republic of Iran, in both governmental and non-governmental organizations. There w no consensus on the panel size for Delphi studies. So, considering the availability of experts, about 30 participants including researchers, experts, professors, managers, and stakeholders related to the field of nutrition and food policy were recruited. The enrolment process was performed based on the suggestions and comments of the principal investigators (i.e. supervisors and co-supervisors of the dissertation, from which these data were extracted), consultations of other experts (through purposive sampling), and participants’ opinions (through snowball sampling). The inclusion criteria were having enough expertise and experience regarding the subject, being involved in policymaking and/or research, and having enough time to answer the questions.

### Round 1

In the first round of the project, we extracted 21 community-based interventions and programs in six categories, aimed to reduce salt, fat, and sugar intake and increase fruit and vegetable intake from our recent systematic reviews, which were parts of a project conducted for MoHME.^11^ These 21 policy options were extracted from six different zones according to the 4Ps social marketing framework (Table 1).^6^

**Table 1:** List of nutritional interventions and policies to reduce the risk of non-communicable diseases

**Policy category**	**Policy options**
Food promotion	
	Incorporating the course of “principles of healthy eating” in the curriculum and textbooks of students to promote their knowledge and information on choosing healthy food
	Controlling or prohibiting advertisements of unhealthy food products (e.g., those high in fat, salt, and sugar) for the public, especially for children and adolescents
	Promoting community nutrition education about choosing healthy food, disadvantages of high-sugar, high-salt, and high-fat food, and the benefits of increasing fruit and vegetable consumption through information campaigns
	Nutrition education about the disadvantages of unhealthy fats (e.g., saturated fatty acids and trans-fatty acids), healthy food choices at schools, workplace, and in religious communities through on-the-job training and counseling, distribution of posters and pamphlets, or use of email and text messages
Food reformulation	
	The precise control of nutrient content of processed food and revision of the national standards in food production
	Incentive supportive policies and government measures by the food industry to design guidelines for food reformulation
	Replacing saturated fatty acids with unsaturated fatty acids and eliminating trans-fatty acids
	Reformulating high–salt and high-sugar food to reduce the salt and sugar amounts
	Producing healthy substitutes for fat, salt, and sugar in food without affecting their texture and taste
	Reducing the salt content of bread
Food labeling	
	Standard labeling of fat content, saturated and trans-fatty acids, salt, and sugar in food products in order to facilitate consumers’ understanding and literacy
	Providing nutritional information on the content and amount of oil, salt, and sugar in the food served in restaurants and cafeterias
School and workplace food environment regulation	
	Regulating and monitoring the sale of processed and high-fat food; banning food high in trans-fatty acids, sugary beverages, and salty snacks; increasing access to fruits and vegetables in school buffets and canteens at the workplace for adults
	Replacing sugar-sweetened beverages with healthy drinks such as water and milk for children and adolescents in schools
	Placing water dispensers and disposable glasses, and installing motivational messages in place to reduce consumption of sugar-sweetened beverages in schools and workplaces
Fiscal policy	
	Free distribution or decreasing fruit and vegetable prices at schools or workplace
	Increasing taxes on high-fat, high-sugar, and salty food
	Providing subsidies for fruits and vegetables to reduce its price
	Sending free or low-price low-calorie healthy drinks at home
Food supply chain, trade, and investment	
	Supporting and promoting home gardens by educating the principles of cultivating and harvesting products as well as allocating resources
	Applying supportive policies to prevent seasonal changes in the price of fruits and vegetables by increasing the infrastructure necessary for storing fruits and vegetables
	Revising the agricultural policies and food supply levels, and establishing cropping patterns based on the provision of standard nutrients, considering the ecological capacities of the country and the sustainability of food production

According to our classification, we designed a specific questionnaire consisting of six main policy option categories. The panel of experts was asked to rate policy options with regard to seven factors (i.e. impact rate of policy option in preventing NCDs, implementation feasibility, implementation costs, chance of stability, acceptance by the authority and society, and other beneficial side effects of policies for non-health domains) on a 5-point Likert scale scoring (1 = Very low, 2 = Low, 3 = Moderate, 4 = High, and 5 = Very high). For example, if they think the policy option had a high impact in terms of preventing NCDs, it is rated 5; if it has low importance, it is scored 1.

Two open-ended questions were asked about the suggested policy options that were missing in the list. In addition, the participant’s opinion was inquired, regarding the best type of integration between these policy options to prevent NCDs.

### Round 2

After collecting and analyzing the questionnaire of round 1, the quantitative summary of the combined responses from each policy option was calculated as the indicator of consensus. Moreover, the policy options suggested by the participants were added to the second-round questionnaire. Next, the questionnaire was sent to all the participants in case they desired to change their opinion scoring regarding the mean score of each policy option. Eventually, all the participants confirmed the prioritized policies at the end of round 2, which suggested the consensus.

### Statistical Analysis

Data were analyzed using basic descriptive statistical tests and expressed as mean, median, interquartile rate (IQR), and dispersion index. Dispersion index was calculated through dividing variance by mean in each round. For calculating the score of each policy option, the scores of seven factors/indices for each policy options were summed and divided by 7. An IQR < 1 was used to indicate consensus. Also, the highest mean and lowest dispersion index indicated an option as high priority.

**Results**

The general characteristics of the participants are presented in Table 2. Participants of this research were experts, policymakers, and pundits of the “Nutrition Improvement Office”, economists and agricultural specialists, stakeholders from food industry, the parliament, the media, as well as nutrition and food researchers, “Non-Communicable Disease Management Department” in MoHME, “Middle-Aged and Elderly Health Promotion Department” in MoHME, “Nutrition Departments” in MoHME, health promotion experts in universities, health economists, non-governmental organizations, and directors of the agriculture sector. The questionnaire of round 1 was sent to 30 participants, of which only 23 completed it. Finally, in the second round, 21 participants completed the questionnaire. As mentioned earlier, 21 policy options were extracted from six different zones according to the 4Ps social marketing framework and one policy option was added in the second round, as suggested by most of the participants (Table 1).^6^

**Table 2:** General characteristics of the participants per Delphi survey rounds

**Characteristics**	**Completed round 1 (n=23)**	**Completed round 2 (n=21)**
Gender		
Male	15	13
Female	8	8
Job position(n)		
Food industry	2	2
Parliament	1	1
The Media	1	1
Nutrition and food researchers	4	4
“Non-Communicable Disease Management Department” in MoHME	4	4
“Middle Aged and Elderly Health Promotion Department” in MoHME	2	2
“Nutrition departments” in MoHME	2	2
Health promotion experts in university	1	1
Health economist	2	2
Directors of the agriculture sector	2	1
NGO	2	1

Abbreviations: MoHME, Ministry of Health and Medical Education; NGO, Non-governmental organization.

**Table 3:** Priority of policy category with regard to participants’ consensus

**Policy category**	**Mean**	**SD**	**IQR**	**Variance-to-mean ratio**
Food reformulation	4.44	0.60	0.69	0.08
Food promotion	3.82	0.42	0.57	0.04
Food labeling	3.65	0.37	0.46	0.03
School and workplace food environment regulation	3.42	0.39	0.67	0.04
Food supply chain, trade, and investment	3.39	0.50	0.68	0.07
Fiscal policy	3.26	0.53	0.61	0.08

The policy options are prioritized according to the highest mean and lowest dispersion index. The first five highest priority policy options concluded by experts were (1) “Incorporating the course of ‘principles of healthy eating’ in the curriculum and textbooks of students to promote knowledge and information on choosing healthy food” with the highest mean (4.12 ± 0.31) and lowest dispersion index (0.02)); (2) “Promoting community education and customizing healthy food choice, educating the consumers on the detrimental effects of high fats, sugar, and salt intake, and increasing fruit and vegetable intake by holding campaigns”; (3) “Reformulation of food products to replace saturated fatty acids with unsaturated fatty acids and eliminating trans-fatty acids”; (4) “Standard labeling on fat, saturated and trans-fatty acids, and sugar and salt content of food based on the consumers’ understanding”; and (5) “Reformulating high-salt and high-sugar food in order to reduce the salt and sugar amounts”. The lowest policy priority option with the lowest mean (2.88 ± 0.86) and highest dispersion index (0.2) was “Sending free or low-price low-calorie healthy drinks at home”. In the policy category analysis, policy options belonging to “changing food content”, “nutrition education”, and “food labeling” were mentioned as the highest priorities of the participants, respectively (Table S1; see Supplementary file 1).

In round 1, most of the participants suggested that “Incorporating the course of ‘principles of healthy eating’ in the curriculum and textbooks of students in order to promote knowledge and information for choosing healthy food” is a necessary policy option to prevent NCDs in Iran. This policy option was added to the policy lists in round 2 of the survey. In response to the question on the best type of integration of policy options, most of the experts recommended that integration of nutrition education with into food labeling and food reformulation could result in fruitful outcomes. Moreover, in terms of fiscal policy, they indicated that, to reach favorable results, taxation on unhealthy food should be accompanied by providing subsidies on the healthy food.

## Discussion

Findings of the present study offer some practical priorities and stances of nutritional policy options for controlling NCD in Iran. This study was done for the first time in order to prioritize nutritional policy options in NCD control field and could be considered as a pilot study for other works in this realm of public health. The experts who participated in this study were more interested in information promotion and became more surprised. It is of note that the experts were from different organizations.

According to the findings of the present study, enhancing consumers’ knowledge, reformulating food production to reduce fat, sugar, salt contents, and food labeling were the highest prioritized policies for declining the burden of NCDs in Iran. Among the policies for enhancing consumers’ knowledge, the category of supplementary lessons on healthy nutrition at schools and universities had the highest importance. In a recent study by Chobandmowlae et al in Iran, which was conducted based on Delphi methods for prioritizing policies for childhood obesity prevention, health experts concluded that incorporating lessons related to a healthy diet at schools was the best policy for dealing with obesity in children.^12^

These results show that most of the experts involved in this field understood the necessity of a rising public nutrition awareness as a feasible and cost-effective tool that could simply be implemented in Iran, with no need for sophisticated procedures. Nevertheless, the government and society might have higher acceptance toward different types of information enhancing programs such as the mass media campaign or poster distribution.

Schools and other educating environments are the main targets of nutrition education worldwide because children spend most of their time at schools and most of the individual food habits develop in the school environment and during childhood. Furthermore, children are an important connection between school and family, so any education in these regards could affect all other members of the family as well. Although several countries such as China, Argentina, England, and South Africa have included nutrition education in the elementary and secondary school curriculum,^13,14^ it seems that nutrition education at schools has not yet found its place in the world. In this regard, insufficient time is devoted to this issue and only gradual progress with insufficient time is seen in this respect. The primary results of Nutrition Education in Basic Schools project of Zambia indicated an improvement in children’s knowledge, attitude, and behavior toward healthy eating. The progress observed in this project was achieved via an active nutrition education program.^15^ In Iran, similar to other countries, there is no comprehensive nutrition education program at schools, such that only few interventional nutrition education programs have had positive effects on healthy dietary choices. Nevertheless, due to the cross-sectional methodology of the studies, short duration, and small sample size, conflicting results have been obtained. Hence, it is necessary to conduct long-term studies with educational programs to achieve desirable results.^16-19^ In this regard, school-based nutrition education should emphasize the development of skills and behaviors related to food preparation and storage that are conducive to healthier food choices.^20^

In our study, promoting public nutrition education and customizing healthy food choices by holding campaigns and advertisements were the second high-priority policy (3.87 ± 0.57). Public health education is one of the main perspectives of propagating healthy food choices and controlling NCD. Campaigns with the aim of enhancing public knowledge have reported increased intake of fruits and vegetables, and reduced salt intake. In Pakistan, between years 1999 and 2004, distributing papers that carried messages in favor of increased fruit and vegetable intake led to a 40% increase in fruit and vegetable intake among 75% of individuals who read the newspaper regularly. In the United States (1995), fruit and vegetable intake increased from 3.4 serving to 4.1 serving per day after publishing 6 months of computer-based newsletters.^21^ A mass media campaign on informing people to reduce their salt intake in Finland resulted in a 53.5% reduction in salt intake (reduced from 14 g in 1997 to 9 g in 2002).^22^ In Iran, in the National Health Week, several information campaigns such as “Watch Out for Your Blood Pressure” in 2018 were held for one month to enhance awareness on hypertension through emphasizing the importance of salt intake for blood pressure control.^23^ In developing countries such as Iran, these types of programs are considered as the most efficient interventions because the most important risk factor for NCDs is insufficient knowledge about modifiable risk factors of these diseases. Furthermore, necessary infrastructure, technology, and resources are lacking in other policy fields such as “changing the content of food” and “producing alternatives for unhealthy food”. Accordingly, it can be concluded that if education is developed and administered in relation to the target group’s needs and characteristics, it will be one of the most feasible, cost-effective, and practical policies.^24^

Considering various numbers of policy options in each policy group, the mean score of the proposed policies in each group was considered, as well. According to our findings, changing the content of food, enhancing consumers’ knowledge, and food labeling were introduced as the highest priorities, respectively (Table 3).

Governments have gradually decreased the trans-fatty acids, aiming to reach a zero content of trans-fatty acids in food. Denmark banned partially hydrogenated oils in 2003 and, in 2006, New York City passed such a ban for restaurant food.^25,26^ This policy will be one of the most efficient policies for eliminating trans-fatty acids from the diet if it had administered properly in the countries. Nonetheless, food industries usually resist these types of policy options. This is mainly due to that point that total elimination of fatty acids requires introducing alternative fatty acids with a novel formulation. Among the best procedures for reducing fat contents, saturated fatty acids, and trans-fatty acids in the food industry are altering fat composition by genetic and chemical manipulating of food through hydrogenation.^27,28^ In a few countries, food industries have commenced their regulating programs according to the market authority in order to prevent the government’s specific regulations. In fact, enhancing consumers’ information about the nutrient compositions of packed food has provoked producers to voluntarily reformulate their products. Under other circumstances, implementing governmental guidelines and regulations may be needed in applying industrial regulations.^29^

In the present study, the participants believed that food reformulation policy category had the highest priority in preventing NCDs. However, it is noteworthy that this type of policy and program needs vast research cost, infrastructure, training, and guidelines for industries and also takes a long time to be accepted by the public and food industry stakeholders.

Food labeling, which allows for the clarification of nutritional characteristics of food, was the third policy priority extracted in the present research. Fatty acid labeling, especially for trans-fatty acids, has voluntarily or compulsory been implemented in several countries. In this regard, the Canadian government has obliged companies to report the trans-fatty acid content of food in food labeling, which would ultimately lead to a reduced amount of trans-fatty acid in food. In 2002, the trans-fatty acid content of food was calculated by subtracting the amount of saturated and unsaturated fatty acids from the total fat content. In 2006, the trans-fatty acid content of food was specifically labeled on food products. According to Ricciuto et al, the mean content of trans-fatty acids and monounsaturated fatty acids of margarine was reduced and the amount of polyunsaturated fatty acid increased in 2006 compared with 2002. This reformulation was the result of the governmental enforcement on factories to report the trans-fatty acid content of their products. As a result, it encouraged food industries to reduce the trans-fatty acid content of products. Consequently, the number of margarine brands with trans-fatty acids content of less than or equal to 0.2 g per serving increased from 31% in 2002 to 69% in 2006.^30^ It has been reported that although the margarine available in markets has become healthier, these changes have increased its cost, making low-income population unable to purchase these products. The compulsory labeling and voluntary restrictions in Canada resulted in a 35% reduction in the trans-fatty acid content of breast milk and a 30% reduction in dietary intake of trans-fatty acids. However, in the United States, compulsory food labeling resulted in a 58% reduction in plasma trans-fatty acids. In addition, it resulted in a decrease in plasma cholesterol and triglyceride and increase in high-density lipoprotein cholesterol.^29^ Despite the mentioned advantages, in societies with low socio-economic status, the net effect of labeling policies relies on individuals’ knowledge regarding food labeling. Thus, implementing labeling along with other interventions such as holding educational campaigns, providing reasonable prices, and improving food standards is advisable.^21^ For instance, in a recent purchase study in Tehran regarding food labeling, consumers with higher education and income were more aware of food label information when purchasing food products.^31^

## Conclusion

Development and implementation of comprehensive NCD policy are sophisticated. In this regard, many factors including the politics, economic situation, social structure, legal system, values of a people, and influence of the interest groups and institutions affect policymaking. In Iran, like other developing countries, to offer more precise prioritization, it is necessary to establish the cost, value, and feasibility of implementing these policies.

Moreover, it is essential to provide sub-structures and conduct research in the food industry to provide appropriate alternatives and methods for changing the content of food and reducing the amount of unhealthy nutrients in food products. We hope that the results of the present research be useful for policymakers to take necessary measures for subsequent enhancement of food choices of the Iranian population.

## Ethical approval

This study was approved by Ethics Committee in Shahid Beheshti University of Medical Sciences (Ethics No. IR.SBMU.NNFTRI.1397.056).

## Competing interests

The authors declare that they have no competing interests.

## Authors’ contributions

HEZ, SP, and NK were involved in designing the research, conducting the research steps, performing data analyses, and drafting the manuscript. AO and JST were involved in conducting the research steps and making the sampling and interpretation on the study results.

## Funding

We kindly acknowledge National Nutrition and Food Technology Research Institute, Faculty of Nutrition Sciences and Food Technology, Shahid Beheshti University of Medical Sciences, for their financial support.

## Acknowledgments

Our special thanks go to the experts who participated in this research. This article was extracted from Pourmoradian’s PhD dissertation.

## Supplementary Materials


Supplementary file 1 contains Table S1.
