# Supplementary material for: A Delphi study for exploring nutritional policy priorities to reduce prevalence of non-communicable diseases in Islamic Republic of Iran
Source: Health Promot Perspect. 2019 Aug 6;9(3):241–7. doi: 10.15171/hpp.2019.33 (PMC6717923; doi:10.15171/hpp.2019.33)
Supplement: Supplementary file 1 — contains Table S1. [file hpp-9-241-s001.pdf]

Supplementary file 1

Table S1. Prioritizing policy options with regard to the mean and variance to mean ratio in rounds 1 and 2

Policy priority	Policy option	Scores in first round				Scores in second round			
		**Mean±SD**	**Variance / mean ratio**	**median**	**IQR**	**Mean ±SD**	**Variance/ mean ratio**	**media n**	**IQR**
1	Incorporating the course of “principles of healthy eating” in the curriculum and textbooks of students to promote their knowledge and information on choosing healthy food	-	-	-	-	4.12±0.31	0.02	4.14	0.43
2	Promoting community nutrition education about choosing healthy food, disadvantages of high-sugar, high-salt, and high-fat food, and benefits of increasing fruit and vegetable consumption through information campaigns	3.91±0.51	0.06	4	0.71	3.87±0.57	0.07	3.92	0.71
3	Replacing saturated fatty acids with unsaturated fatty acids and eliminating trans-fatty acids	3.89±0.60	0.09	3.85	0.75	3.84±0.63	0.10	3.85	0.89
4	Standard labeling of fat content, saturated and trans-fatty acids, salt, and sugar in food products in order to facilitate consumers’ understanding and literacy	3.83±0.35	0.03	3.85	0.57	3.77±0.41	0.04	3.85	0.61
5	Reformulating high–salt and high-sugar food to reduce the salt and sugar amounts	3.81±0.59	0.09	3.85	0.79	3.75±0.63	0.10	3.78	0.93
6	Producing healthy substitutes for fat, salt, and sugar in food without affecting their texture and taste	3.78±0.43	0.05	3.85	0.75	3.73±0.48	0.06	3.78	0.75
7	Nutrition education about the disadvantages of unhealthy fats (e.g., saturated fatty acids	3.81±0.58	0.09	3.92	0.61	3.72±0.61	0.10	3.78	0.61

	and trans-fatty acids), healthy food choices at schools, workplace, and in religious communities through on-the-job training and counseling, distribution of posters and pamphlets, or use of email and text messages								
8	Precise control of nutrient content of processed food and revision of the national standards in food production	3.81±0.66	0.11	3.92	0.71	3.70±0.67	0.12	3.85	0.79
9	Reducing salt contents of bread	3.75±0.55	0.08	3.78	0.75	3.69±0.59	0.09	3.78	0.86
10	Revising the agricultural policies and food supply levels and establishing cropping patterns based on the provision of standard nutrients, considering the ecological capacities of the country and the sustainability of food production	3.62±0.50	0.07	3.42	0.75	3.59±0.51	0.07	3.42	0.71
11	Controlling or prohibiting advertisements of unhealthy food products (e.g., those high in fat, salt, and sugar) for the public, especially for children and adolescents	3.57±0.56	0.08	3.92	0.61	3.55±0.60	0.10	3.78	0.61
12	Providing nutritional information on the content and amount of oil, salt, and sugar in the foods served in restaurants and cafeterias	3.54±0.43	0.05	3.57	0.61	3.53±0.42	0.05	3.50	0.71
13	Incentive supportive policies and governmental measures by the food industry to design guidelines for food reformulation	3.57±0.62	0.11	3.57	0.71	3.51±0.61	0.10	3.57	0.75
14	Applying supportive policies to prevent seasonal changes in the price of fruits and vegetables by increasing the	3.57±0.69	0.13	3.57	1	3.51±0.66	0.12	3.57	0.82

	infrastructure necessary for storing fruits and vegetables								
15	Replacing sugar-sweetened beverages with healthy drinks such as water and milk for children and adolescents in schools	3.58±0.41	0.04	3.71	0.64	3.50±0.43	0.05	3.64	0.71
16	Increasing taxes on high-fat, high-sugar, and salty food	3.46±0.53	0.13	3.28	0.71	3.42±0.56	0.09	3.35	0.57
17	Free distribution or decreasing fruit and vegetable prices at schools or workplace	3.44±0.67	0.12	3.28	0.71	3.42±0.70	0.14	3.57	0.90
18	Regulating and monitoring the sale of processed and high-fat food; banning food high in trans-fatty acids, sugary beverages, and salty snacks; increasing access to fruits and vegetables in school buffets, and canteens at the workplace for adults	3.42±0.48	0.06	3.28	0.86	3.40±0.47	0.06	3.28	0.86
19	Placing water dispensers and disposable glasses and installing motivational messages in place to reduce consumption of sugar-sweetened beverages in schools and workplaces	3.41±0.51	0.07	3.42	0.61	3.37±0.52	0.08	3.42	0.64
20	Providing subsidies for fruits and vegetables to reduce its price	3.53±0.66	0.13	3.28	0.71	3.32±0.67	0.13	3.28	0.57
21	Supporting and promoting home gardens by educating the principles of cultivating and harvesting of products as well as allocating resources					3.07±0.63	0.13	3	0.89
22	Sending free or low-price low-calorie healthy drinks at home	2.95±0.74	0.18	3	0.61	2.88±0.86	0.20	2.78	0.68
